# Selective inhibition of electrical conduction within the pulmonary veins by α1-adrenergic receptors activation in the Rat

**DOI:** 10.1038/s41598-020-62349-5

**Published:** 2020-03-25

**Authors:** Pierre Bredeloux, Ian Findlay, Côme Pasqualin, Mélèze Hocini, Olivier Bernus, Véronique Maupoil

**Affiliations:** 1 EA7349, Laboratoire STIM, Groupe Physiologie des Cellules Cardiaques et Vasculaires, Université de Tours, Tours, France; 2 IHU Liryc, Electrophysiology and Heart Modeling Institute, Fondation Bordeaux Université, Bordeaux, France; 3 Université de Bordeaux, Centre de Recherche Cardio-Thoracique de Bordeaux, INSERM U1045 Bordeaux, France; 4 Bordeaux University Hospital (CHU), Cardiac Electrophysiology and Cardiac Stimulation Team, Bordeaux, France

**Keywords:** Diseases, Cardiovascular diseases, Cardiology, Cardiovascular biology, Medical research, Preclinical research

## Abstract

Pulmonary veins (PV) are involved in the pathophysiology of paroxysmal atrial fibrillation. In the rat, left atrium (LA) and PV cardiomyocytes have different reactions to α1-adrenergic receptor activation. In freely beating atria-PV preparations, we found that electrical field potential (EFP) originated from the sino-atrial node propagated through the LA and the PV. The α1-adrenergic receptor agonist cirazoline induced a progressive loss of EFP conduction in the PV whereas it was maintained in the LA. This could be reproduced in preparations electrically paced at 5 Hz in LA. During pacing at 10 Hz in the PV where high firing rate ectopic foci can occur, cirazoline stopped EFP conduction from the PV to the LA, which allowed the sino-atrial node to resume its pace-making function. Loss of conduction in the PV was associated with depolarization of the diastolic membrane potential of PV cardiomyocytes. Adenosine, which reversed the cirazoline-induced depolarization of the diastolic membrane potential of PV cardiomyocytes, restored full over-shooting action potentials and EFP conduction in the PV. In conclusion, selective activation of α1-adrenergic receptors results in the abolition of electrical conduction within the PV. These results highlight a potentially novel pharmacological approach to treat paroxysmal atrial fibrillation by targeting directly the PV myocardium.

## Introduction

Atrial fibrillation (AF), the most common arrhythmia in human, is in its paroxysmal stage, mainly induced by ectopic activity arising from the pulmonary veins (PV) myocardial sleeves^1^. Antiarrhythmic drugs used to treat AF are poorly efficient and present a high risk of adverse events. To cure patients with symptomatic AF refractory or intolerant to antiarrhythmic drugs, PV isolation by intra-cardiac catheter ablation is recommended^2^. However, despite a high success rate, this intervention only benefits a small proportion of patients due to the sheer number of AF sufferers worldwide. In addition, arrhythmia recurrence related to PV reconnections are commonly observed and require additional interventions^3–5^. Therefore, new pharmacological treatments are necessary. To achieve this objective, investigations have mainly been directed towards drugs acting at the atrial level but unfortunately without any success^6,7^. No successful attempts have been made so far towards the development of PV-specific drugs.

In rat PV strips^8,9^, norepinephrine has a biphasic effect on the cardiomyocytes resting membrane potential consisting of an initial hyperpolarization followed by a slow depolarization, which precede the appearance of an automatic activity. The selective activation of α1-adrenergic receptors reproduces the depolarization induced by norepinephrine and leads to cardiomyocytes in-excitability^8^. This is not observed in left atria (LA) strips.

The precise physiological role of α1-adrenergic receptors in PV cardiomyocytes is not fully understood but our previous observations allow us to consider the possibility of inhibiting the conduction of PV ectopic activity to the LA using a pharmacological approach. Moreover several studies showed that these receptors are present and functional in the human myocardium^10,11^ suggesting that this strategy might be transposed to humans. This attractive prospect, led us to investigate the effects of the α1-adrenergic receptors activation on the electrical conduction between PV and the atria isolated from rats by means of electrical mapping realized with a linear multiple extracellular electrode array (LMEA).

## Results

### Electrical conduction within the PVs-atria preparations in sinus rhythm

**Figure 1:**
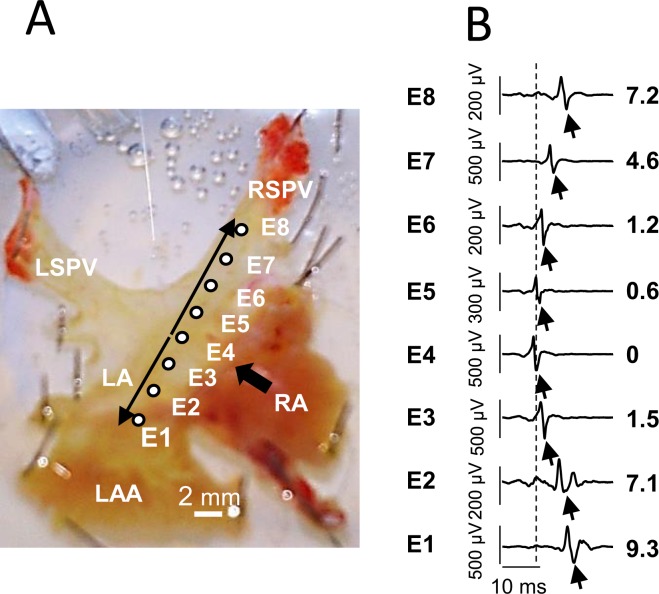
(**A**) The electrode array position for recording from the LA (E1 to E4) along the right superior PV (RSPV, E5 to E8) on a representative preparation (LSPV and RSPV: left and right superior pulmonary vein, LA and RA: left and right atria, LAA: left atria appendage). (**B**) An example of EFP recordings obtained from an array positioned as shown in A under basal conditions and during spontaneous sinus rhythm (330 bpm). The activation time for each electrode (ms) relative to E4 which was the first that detected a signal (indicated by arrows) is shown to the right.

Electrical conduction was recorded through EFPs in 10 spontaneously beating PVs-atria preparations (286 ± 12 beats/min) with an 8 electrodes LMEA. A representative preparation and EFP recording is shown in Fig. 1. The earliest EFP signal was recorded on E4 (black arrow, Fig. 1A) at the junction between the PVs ostium and LA roof. The EFP delays showed that electrical activity then spread along the PV (from E4 to E8) and towards the LAA (from E4 to E1). In our experimental conditions, the estimated conduction velocity was similar within the LA and the PV *i.e*. about 1 m.s^−1^.

### α1-adrenergic receptor activation selectively suppresses electrical activity within pulmonary veins

**Figure 2:**
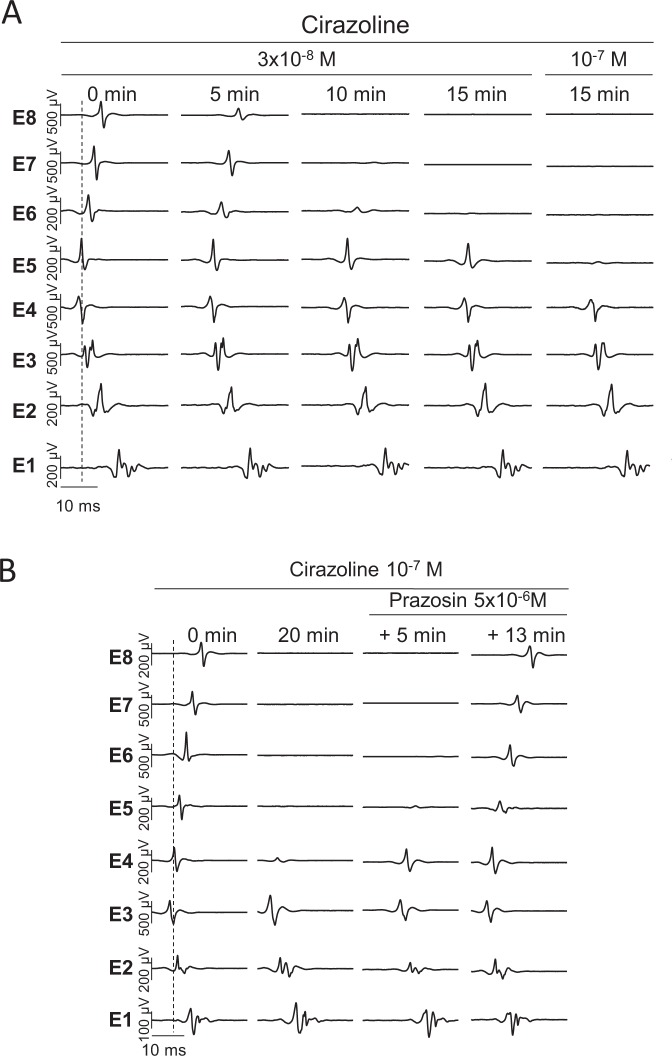
(**A**) A representative recording of electrical activity in a preparation driven by sinus rhythm during the superfusion of 3 × 10^−8^ and 10^−7^ M cirazoline. The position of the array was that shown in Fig. 1A. Conduction was lost first in the distal PV (E8) and then progressively down the length of the vein (E7 to E5). Conduction across the LA (E4 to E1) was maintained. (**B**) Reversal of the effect of cirazoline with prazosin. In a different preparation driven by sinus rhythm, the superfusion of 10^−7^ M cirazoline had led to the loss of conduction along the PV (E4 to E8) while retaining conduction across the LA (E4 to E1). The addition of 5 × 10^−6^ M prazosin in the continued presence of cirazoline, led to the progressive recovery of conduction along the PV from its base (E4) to its distal part (E8).

In these 10 spontaneously beating PVs-atria preparations, the selective α1-adrenergic receptor agonist cirazoline (3 × 10^−8^ then 10^−7^ M) induced a time-dependent loss of electrical activity both in the right and in the left superior pulmonary vein of the rat (see Fig. 2 and Fig. S1 in the Supplementary Data). This effect began between 3 and 10 min (mean 7.4 ± 1.7 min) in the distal region of the PV (E8) and then extended to its base (E4) 15 minutes after the onset of cirazoline superfusion. At the same time electrical conduction was maintained from the LA roof toward the LAA (E4 to E1) (Fig. 2A). A higher concentration of 10^−5^ M cirazoline did not have any additional effect (data not shown). This loss of electrical conduction within the PV was dependent upon α1-adrenergic receptor activation since the addition of the selective α1-adrenergic receptor antagonist prazosin (5 × 10^−6^ M), in the continued presence of cirazoline, progressively restored conduction along the PV from its base in 6.5 ± 1.1 min (E4) to its distal part (E8) in 15.5 ± 1.3 min (Fig. 2B, n = 6).

### α1-adrenergic receptor activation decreases conduction velocity within the pulmonary vein

The next series of experiments were conducted in electrically paced (5 Hz) PVs-LA preparations to avoid the effect of spontaneous sinus drive frequency variation on conduction velocity in different preparations. Stimuli were applied to the apex of the LAA.

**Figure 3:**
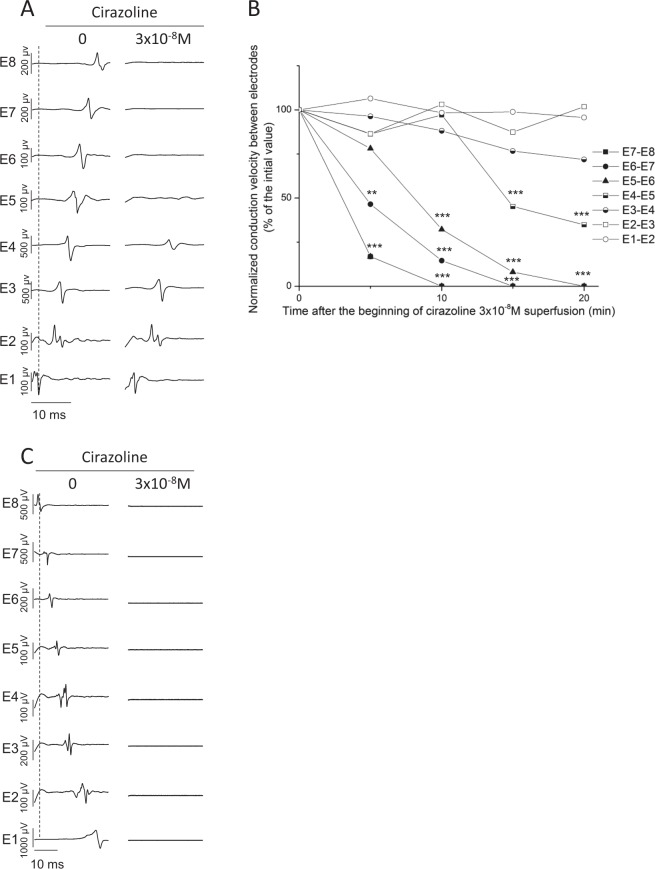
(**A**) Conduction across the LA and along the PV in a preparation driven by 5 Hz electrical stimulation applied to the base of the LAA. In 3 × 10^−8^ M cirazoline conduction was maintained across the LA (E1 to E4) while conduction along the PV (E5 to E8) was lost. (**B**) Effect of cirazoline upon the conduction velocity of electrical signals evoked by stimulation in the LAA. The application of cirazoline led to the progressive reduction of conduction velocity (measured as the time taken for the signal to pass between 2 adjacent electrodes) prior to the loss of the electrical signal between E5 to E8 in the distal PV. This decrease was less pronounced at the junction between PV and LA (between E5 to E3) and was not observed in the LA (between E3 to E1) (n = 5, **p < 0.01, ***p < 0.001 vs 0 min, standard error bars have been omitted for clarity). (**C**) When electrical stimulation was applied to the distal PV, cirazoline (3 × 10^−8^ M) blocked conduction not only along the vein (E8 to E5) but also across the LA (E4 to E1).

In each of 5 preparations, EFP were recorded first in E1, localized in the LAA, and last in E8 localized in the distal part of the PV indicating electrical conduction from the LAA, across the LA and along the PV (Fig. 3A). Cirazoline (3 × 10^−8^ M) induced a time-dependent loss of electrical activity which began in the distal part of the PV (E8) after 5 ± 2 min and progressively extended to its base (E5) after 41 ± 6 min of superfusion. The conduction from the base of the LAA (E1) to the LA (E4) was maintained (Fig. 3A). Prior to this loss of electrical activity, cirazoline induced a progressive decrease of the conduction velocity of electrical signal between each electrode positioned on the PV (E4 to E8) (Fig. 3B). Thus, the decrease of the conduction velocity first begun at the distal part of the PV and then progressed to its base at the junction with LA. Conduction velocity in the LA roof was not affected (E4 to E1).

In another set of experiments (n = 5), PV-LA preparations were electrically paced from stimulation wires placed on the distal part of the PV. At a frequency of 5 Hz, activation time increased from E8 positioned at the distal part of the PV to E1 positioned in the LA. Superfusion of cirazoline (3 × 10^−8^ M) completely abolished the electrical activity in the PV and its conduction to the LA (Fig. 3C). It was noted that the time required to abolish activity in the PV in these preparations (18 ± 2.7 min) was significantly shorter than the time required to obtain complete PV isolation when preparations were paced in the LAA (p = 0.008). In these experimental conditions, we did not observe any modification of conduction velocity prior to the loss of electrical activity.

### The loss of electrical conduction induced by α1-adrenergic receptor activation within the pulmonary veins is due to cardiomyocyte depolarization

**Figure 4:**
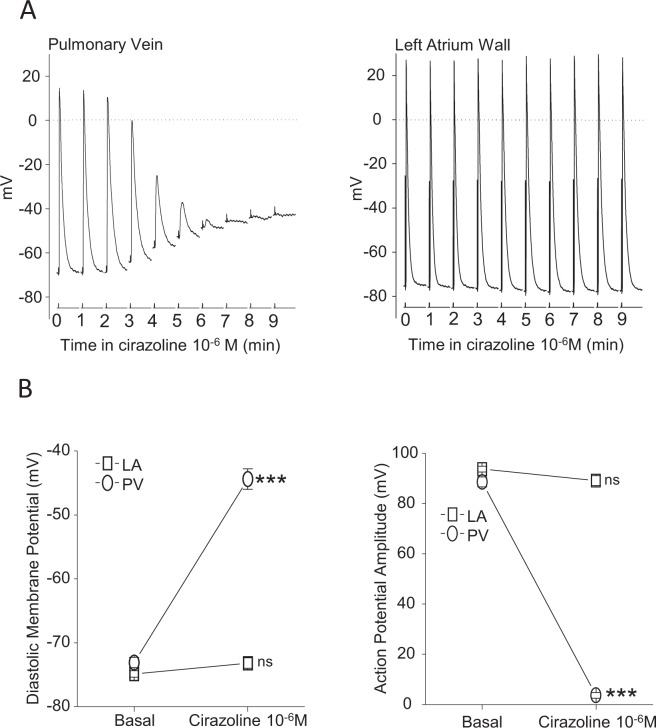
(**A**) Simultaneous intracellular microelectrode recordings from the PV (left) and the LA (right) in one preparation during the superfusion of 10^−6^ M cirazoline. Pairs of AP are shown at one minute intervals from an otherwise continuous recording stimulated at 5 Hz. (**B**) The effects of 10^−6^ M cirazoline upon DMP (left) and AP amplitude (right) of electrically evoked AP recorded simultaneously in the LA (square symbols) and PV (round symbols). Symbols represent mean ± SEM of results obtained from 14 different preparations which were electrically paced at 5 Hz in the LA. ***p < 0.001 vs basal conditions. ns: no significant difference vs basal condition.

Simultaneous recordings of LA and PV AP with intracellular microelectrodes were performed to evaluate the electrophysiological modification induced by cirazoline in 14 PVs-LA preparations electrically paced at 5 Hz (Fig. 4). In PV cardiomyocytes, cirazoline induced a time-dependent depolarization of DMP from −73.9 ± 0.9 mV to −45.1 ± 2.1 mV (p < 0.001) that was associated with a progressive decrease of AP amplitude from 91.1 ± 2.2 mV to 5.4 ± 1.6 mV (p < 0.001). At the same time in the LA, cirazoline had no significant effect upon the DMP or AP amplitude, though it increased APD_90_ from 36.3 ± 1.8 ms to 44.7 ± 2.5 ms (p < 0.05). In each preparation, full over shooting APs were still recorded in the LA in the presence of 10^−6^ M cirazoline when little or no signal could be recorded in the PV. This accounts for the preservation of electrical conduction in the LA (Figs. 2 and 3). The main AP parameters obtained in absence and presence of cirazoline in the PV and in the LA are summarized in Table 1.

**Table 1:** Action potential parameters recorded simultaneously in the left atria and the pulmonary veins in basal conditions and at the steady-state of the cirazoline 10^−6^ M superfusion.

	DMP (mV)	AP peak (mV)	APA (mV)	P0 dV/dt_max_ (V/s)	APD90 (ms)
LA basal	−74.4 ± 0.6	18.4 ± 1.0	92.8 ± 1.2	172 ± 10	36.3 ± 1.8
LA cirazoline	−71.6 ± 0.7	14.5 ± 1.0	86.1 ± 1.1	144 ± 12	44.7 ± 2.5*
PV basal	−73.9 ± 0.9	17.6 ± 1.8	91.1 ± 2.2	192 ± 11	46.5 ± 2.6
PV cirazoline	−45.1 ± 2.1***	−39.5 ± 2.0	5.4 ± 1.6***	—	—

DMP (diastolic membrane potential), AP peak (maximum voltage of action potential), APA (action potential amplitude), P0 dV/dtmax (phase 0 maximum rate of depolarization) and APD90 (action potential duration at 90% of repolarization). Mean ± SEM of results obtained from 14 different preparations electrically paced at 5 Hz in the LA. *P < 0.05 and ***p < 0.001 vs basal condition.

### α1-adrenergic receptor activation suppresses EFP conduction from the PV to the LA and restores physiological conduction in preparations in sinus rhythm

**Figure 5:**
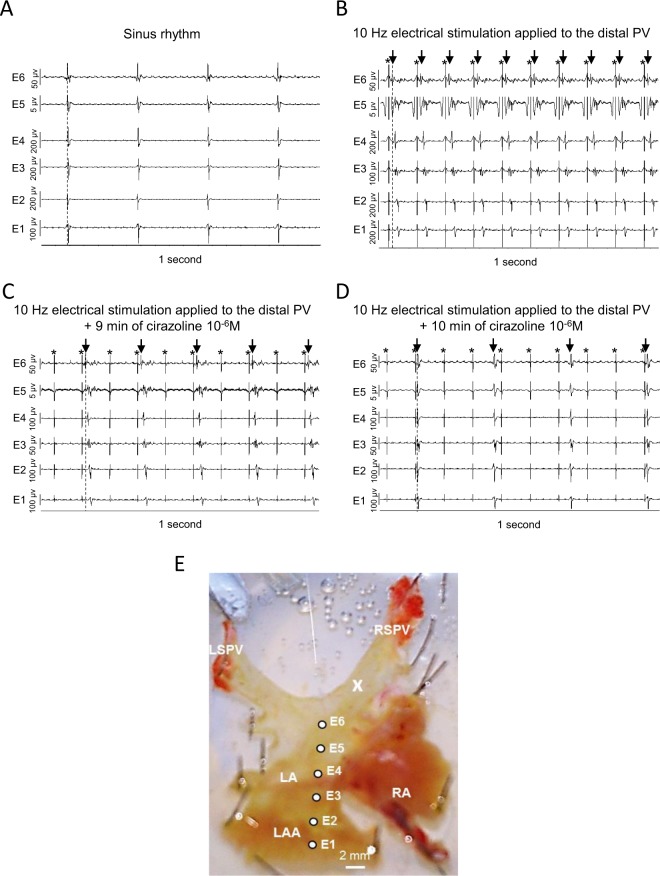
(**A**) A representative recording of electrical activity in the LA of a preparation driven by sinus rhythm from the right atrium (240 beats/min). The position of the electrode array is indicated in (E). (**B**) Conduction across the LA in the same preparation driven by 10 Hz electrical stimulation applied to the distal PV under control conditions. (**C**) After 9 minutes of superfusion of 10^−6^ M cirazoline, electric stimulation to the PV failed to evoke one-to-one EFP and conduction within the LA. (**D**) 1 minute later, the 10 Hz electrical stimulation to the PV failed to drive the preparation and conduction from the sinus node was restored within the LA. Stimulus artifacts and EFP are indicated by stars and arrows respectively in (**B–D**). (**E**) X indicates the position of the stimulation electrodes used in this series of experiments.

Considering these last results, we used the 6 electrodes LMEA to record electrical conduction in the LA of 7 spontaneously beating PVs-atria preparations (253.5 ± 19.7 beats/min) (Fig. 5A). A representative preparation with the electrodes position is shown in Fig. 5E. These preparations were then paced at 10 Hz in the distal part of the PV where high firing rate ectopic foci can occur in human. In these experimental conditions EFP conduction from the sinus node was no longer observed within the LA and the preparations were driven by conduction of EFP evoked in the PV, from E6 positioned in the LA roof to E1 positioned in the LAA (Fig. 5B). After 10 ± 2 min of cirazoline 10^−6^ M superfusion, electric stimulation failed to induce one-to-one EFP conduction from the PV (Fig. 5C) and 47 ± 11 seconds later, EFP conduction from the PV failed entirely and signals from the sinus node was restored within the LA (Fig. 5D).

### Effect of adenosine upon cirazoline-pretreated pulmonary veins

Finally, the effect of adenosine which has been extensively used in human during PV ablation procedures to unmask dormant conduction was studied.

**Figure 6:**
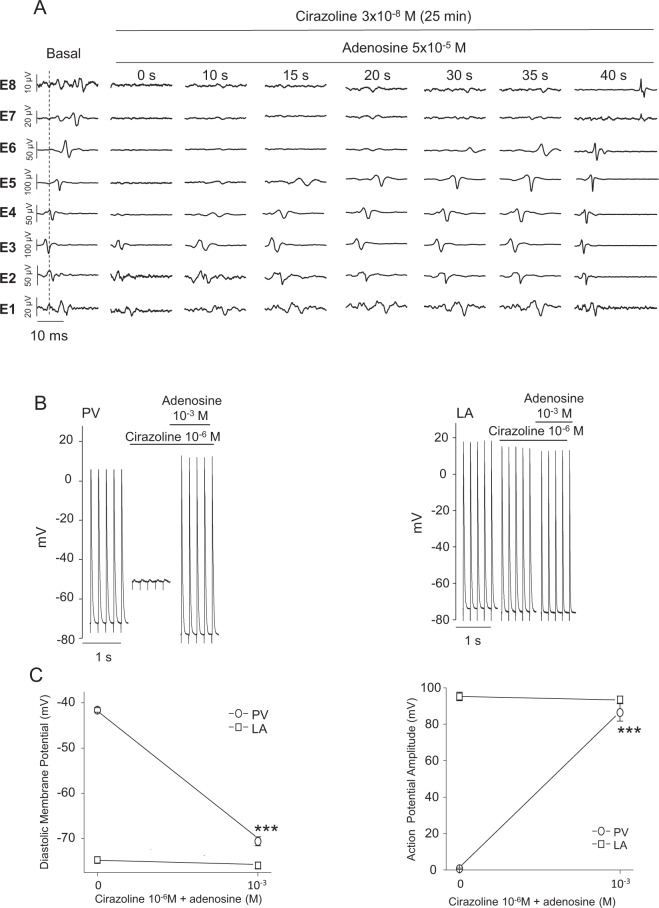
(**A**) The time course of the recovery of conduction in the PV induced by 5 × 10^−5^ M adenosine. In this preparation which was driven by sinus rhythm, conduction in the PV had been lost after 25 min exposure to 3 × 10^−8^ M cirazoline. The addition of adenosine induced the progressive development of conduction along the PV from its base (E4) to its distal part (E8) in 40 seconds. (**B**) Segments of a continuous recording of 5 Hz stimulated AP recorded with intracellular microelectrodes simultaneously from the PV (left) and the LA (right) during the superfusion of 10^−6^ M cirazoline and then the addition of 10^−3^ M of adenosine. (**C**) The effects of 10^−3^ M adenosine upon DMP (left) and AP amplitude (right) of PV (round symbols) and LA (square symbols) AP in the continuous presence of 10^−6^ M cirazoline. Symbols represent mean ± SEM of results obtained from 6 different preparations which were electrically paced at 5 Hz in the LA. ***p < 0.001 vs cirazoline.

In spontaneously beating preparations (214 ± 24 beats/min, n = 7) in which the electrical conduction within the PV was abolished by cirazoline, adenosine (5 × 10^−5^ M) restored electrical conduction in the PV from its base (E4) to its distal part (E8) in 42 ± 11 s (Fig. 6A).

Microelectrode recordings showed that in PVs whose electrical activity has been suppressed by the depolarization induced by cirazoline, the addition of adenosine hyperpolarized the PV cardiomyocyte DMP which resulted in the reappearance of full over-shooting action potentials in the PV myocardium. At the same time, in the LA, in the continued presence of cirazoline, adenosine reduced APD_90_ from 53.5 ± 3.2 ms to 26.6 ± 1.7 ms (p < 0.01; n = 6) (Fig. 6B,C).

Adenosine by itself increased the DMP of PV cardiomyocytes from −72.8 ± 0.8 mV to −76.8 ± 0.7 mV (p < 0.01, n = 7). This effect was associated with an increase of AP amplitude from 87.8 ± 2.0 mV to 100.7 ± 1.1 mV (p < 0.001) and a reduction in APD 90 from 47.1 ± 1.9 ms to 20.4 ± 0.3 ms (p < 0.001). At the same time, in the LA, adenosine by itself had no significant effect upon the DMP (−73.9 ± 0.9 mV *vs*. −74.9 ± 0.9 mV, n = 7) and AP amplitude (92.6 ± 2.5 mV *vs*. 93.6 ± 2.3 mV), though it decreases APD90 from 40.7 ± 3.6 ms to 19.5 ± 1.1 ms (p < 0.001).

## Discussion

In this study, we showed for the first time that α1-adrenergic receptors activation abolishes excitability within PV leading to the loss of electrical conduction without disrupting electrical conduction within the LA.

To perform real-time long-term monitoring of electrical conduction between atria and PV, LMEA made of 6 or 8 recording silver wire electrodes were developed. In fact, commercial multielectrode arrays could not cover the entire area of PV-atria preparation (>20 mm from the base of the LAA to the top of a PV) and do not allow a tight contact between electrodes and tissue to properly record electric signals on all the electrodes. The very high speed of conduction velocity in the rat PV myocardium required electrodes spaced from each other by at least 2 mm (center to center) to visualize the interval between EFP recorded by each electrode positioned along the LA – PV axis. In our experimental conditions, the apparent conduction velocity was about 1 m.s^−1^ in PV and LA which was in accordance with values previously reported in rat atria^12,13^.

The loss of EFP induced by cirazoline in the PV is due to the activation of α1-adrenergic receptors and not to cellular damages induced by the mechanical stretch applied to the preparation since it was completely reversible by the addition of the α1-adrenergic antagonist prazosin. In double microelectrode experiments, cirazoline depolarized the DMP of PV cardiomyocytes, leading to the progressive loss of AP in this tissue. Conversely, in all preparations, DMP as well as conduction were maintained in the LA. This is in accordance with previous results demonstrating that α1-adrenergic receptors activation significantly depolarize the membrane of cardiomyocytes in PV but not in LA strips^8^.

We also showed that cirazoline did not induce a conduction block at the PV-LA junction, but rather a progressive abolition of the excitability of this tissue. This progressive loss of electrical activity in the PV was preceded by a time-dependent decrease in the conduction velocity which began first in the distal part of the PV and then extended to its base. This early effect in the rat PV distal part could be related to the presence of cardiomyocytes with lower negative resting membrane potential (above −50 mV) compared to cardiomyocytes of the proximal PV (about −75 mV) as reported by^14^. This might be due to a variation in the density of certain ionic currents involved in the membrane potential regulation of these cardiomyocytes^15^ and could explain why cirazoline take more time to induce a loss of excitability in the base of PV than in its distal part. This hypothesis is reinforced by the observation that adenosine, by increasing the DMP of cirazoline pretreated PV cardiomyocytes, is able to progressively restore conduction first at the base then at the distal part of PV. In fact, it has been shown that adenosine by activating the G-protein regulated K^+^ current, I_Kado_^16,17^ was able to restore conduction across the depolarized resting membrane potential region induced by an ablation procedure in canine PV^18^.

In atrial cardiomyocytes, I_Kado_ could be inhibited by α1-adrenergic receptor activation^19,20^. In PV cardiomyocytes, the resting membrane potential is less negative than in LA ones^8,21^. This difference could be explained by a lower I_K1_ potassium current^15,21,22^ and a higher Na^+^ basal permeability of the PV cardiomyocytes membrane^23^ compared to LA cardiomyocytes. Thus, a similar inhibition of IKado in PV cardiomyocytes might lead to membrane depolarization in the presence of the background Na^+^ conductance which would no longer be counterbalanced. However, other mechanisms involving the Na^+^/Ca^2+^ exchanger^24^ or chloride channels^25,26^ might also contribute to the depolarization induced by α1-adrenergic receptors activation and will require further investigations.

Finally, the cirazoline-induced loss of excitability, which is specific to PV, suggests that a pharmacological approach to treat AF in an early stage could be possible. Since there is no completely satisfactory animal model of paroxysmal AF originating from the thoracic veins^27^ to test this hypothesis, we paced spontaneously beating preparations at 10 Hz in the PV. In these conditions, we observed electrical conduction from the PV to the LA which was no longer driven by electric signals from the sinus node. Cirazoline prevented activation of PV cardiomyocytes and thus stopped conduction from the PV allowing the return of the physiological conduction in the LA.

In conclusion, this study demonstrates that in the rat, the α1-adrenergic receptors activation induces a selective membrane depolarization of PV cardiomyocytes leading to the loss of excitability, which causes electrical impairment in this tissue. This could offer innovative research perspectives for the development of more targeted pharmacological treatments of paroxysmal AF by preventing the triggering of ectopic electrical activities in the PV. As a proof of concept, we showed that cirazoline restored physiological conduction from the sino-atrial node to the LA in preparations driven by high frequency EFP evoked in the PV. Although α1-adrenergic receptors are expressed and functional in human heart, their activation would obviously lead to systemic adverse events. In prospect, the molecular mechanisms responsible for this depolarization has now to be investigated to find one or more valuable therapeutic targets which will have to be evaluated in human PV.

## Methods

All applicable international, national and/or institutional guidelines for the care and use of animals were followed. All experiments involving animals were approved by the local institutional ethical committee (Comité d’Ethique en Expérimentation Animale Val de Loire, Tours, France. Permit number 2016090711251954).

Male Wistar rats (450 g, CER Janvier, Le Genest St Isle, France) were anesthetized by an intraperitoneal injection of pentobarbital (60 mg/kg). After intravenous injection of heparin (500 UI/kg), the heart-lung block was removed and preparations made up of PV, LA and right atria (RA) were dissected in a dish containing cold cardioplegic solution (in mM: 110 NaCl; 1.2 CaCl_2_; 16 KCl; 16 MgCl_2_; 10 NaHCO_3_; 10 glucose). Experiments were carried out on preparations superfused with Krebs-Henseleit solution (in mM: 119 NaCl; 1.36 CaCl_2_; 4.7 KCl; 1.18 KH_2_PO_4_; 1.17 MgSO_4_; 25 NaHCO_3_; 5.5 glucose; pH 7.4 equilibrated with 95% O_2_ and 5% CO_2_) maintained between 35–37 °C.

### Multi-electrodes array recordings

To record extracellular field potentials (EFP) we used custom-made LMEA consisting of 6 or 8 extracellular electrodes made of PFA-coated silver wire (0.76 mm diameter), arranged in line and spaced apart from each other by 2 mm. Recordings were obtained by placing the LMEA on the right or left superior PV-LA axis (for example see Fig. 1A), with the reference electrode in the saline solution. The recording electrodes, labelled E1 to E8, were connected to a data acquisition system (USB-ME64, Multichannel System, Reutlingen, Germany) connected to a computer running the data acquisition software MC_Rack (Multichannel System, Reutlingen, Germany). Sampling frequency for each signal channel was 20 kHz.

In some experiments, preparations were paced via two shielded Ag/AgCl wires positioned either on the left atrial appendage (LAA) or the distal part of a PV and connected to the Multichannel System stimulus generator, STG4008 (Multichannel System, Reutlingen, Germany), which delivered 1 ms duration biphasic square pulse of current at the frequency of 5 or 10 Hz. For analysis, activation times were determined from the point of the maximal negative slope of each EFP.

### Intracellular recording of electrically evoked action potentials

Electrically evoked action potentials (AP) were recorded simultaneously in the PV (between E7 and E8 in Fig. 1A) and in the LA (close to E2 and E3 in Fig. 1A) with two glass capillary microelectrodes filled with 3 M KCl (20–30 MΩ) connected to a Duo 773 Electrometer amplifier (WPI, Aston, UK). Signals were filtered at 10 kHz low pass, and digitized at a sampling frequency of 40 kHz with a PowerLab 4/25 interface (ADInstruments, Chalgrove, UK). Recordings were acquired on a computer running Chart 5 software. Electrical stimuli consisted of 2 ms duration square wave pulses generated by a Master-9 programmable stimulator (AMPI, Jerusalem, Israel) through a WPI A360 stimulus isolation unit and delivered to the LA via a pair of fine shielded Ag/AgCl wires approximately positioned near E4 in Fig. 1A.

Analysis of AP parameters was performed with the Peak Parameters Module of Chart 5 software (ADInstruments, Chalgrove, UK). Amplitude of AP, duration of the AP at a repolarization of 90% (APD_90_) and the diastolic membrane potential (DMP) were measured at the steady state.

### Statistics

Data are presented as mean values ± SEM, with the number of experiments indicated as n. Statistical significance were assessed using, Mann-Whitney rank sum test, one-way or two-way repeated measure ANOVA (followed by appropriated post-hoc tests) where appropriate (Sigmastat, V3.5, Systat Software Inc, Point Richmond, CA, USA). A P < 0.05 value was considered to be significant.

### Chemicals and reagents

Chemicals used in these experiments were of reagent grade. Norepinephrine, prazosin, and adenosine were obtained from Sigma-Aldrich (Saint Quentin- Fallavier, France) and cirazoline from Tocris (Lille, France). They were prepared as stock solutions in distilled water and kept frozen (−20 °C) until just prior to dilution in saline solution.

## Supplementary information

Supplementary Information.

## Data Availability

All data generated or analysed during this study are available from the corresponding author on reasonable request.
