# Supplementary material for: Selective inhibition of electrical conduction within the pulmonary veins by α1-adrenergic receptors activation in the Rat
Source: Sci Rep. 2020 Mar 25;10:5390. doi: 10.1038/s41598-020-62349-5 (PMC7096464; doi:10.1038/s41598-020-62349-5)
Supplement: Supplementary file 1 — Supplementary Information. [file 41598_2020_62349_MOESM1_ESM.pdf]

Supplementary data

Selective inhibition of electrical conduction within the pulmonary veins by α1-adrenergic receptors activation in the Rat.

Pierre Bredeloux, Ian Findlay, Côme Pasqualin, Mélèze Hocini, Olivier Bernus, and Véronique Maupoil·

Corresponding author:

Pierre Bredeloux : pierre.bredeloux@univ-tours.fr

EA7349, Laboratoire STIM, Groupe Physiologie des Cellules Cardiaques et Vasculaires, Tours, France.

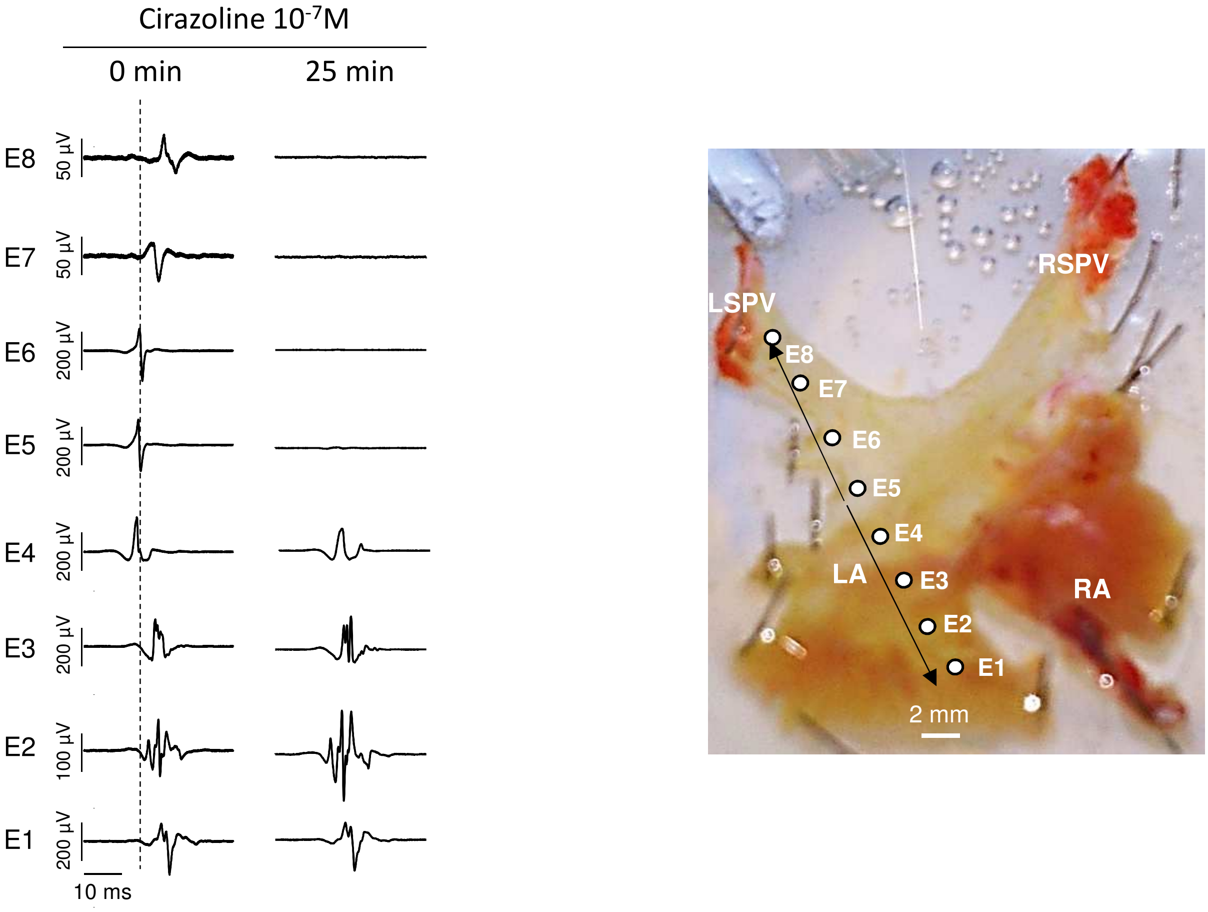

Figure S1 :

Left panel: A representative recording of electrical activity within the left superior pulmonary vein (LSPV) and the left atria (LA) in a preparation driven by sinus rhythm before and 25 min after the superfusion of 10^{-7} M cirazoline. Conduction was lost first in the distal PV (E8) and then progressively down the length of the vein (E7 to E5). Conduction across the LA (E4 to E1) was maintained.

Right panel: The electrode array position on a representative preparation for recording along the LA (E4-E1) and the LSPV (E5-E8).
